# Editorial: The Metaphorical Brain

**DOI:** 10.3389/fnhum.2015.00699

**Published:** 2016-01-05

**Authors:** Seana Coulson, Vicky T. Lai

**Affiliations:** ^1^Cognitive Science Department, University of CaliforniaSan Diego, La Jolla, CA, USA; ^2^Department of Psychology, University of South CarolinaColumbia, SC, USA

**Keywords:** Alzheimer's disease, autism, embodiment, executive function, figurative language comprehension, hemispheric specialization, right hemisphere damage, schizophrenia

Long considered a peripheral topic in linguistics, metaphor is increasingly viewed as a central feature of higher cognition and abstract thought. Investigation of the neural substrate of metaphor has, similarly, become more sophisticated, involving increasingly specific suggestions about the processes involved in its comprehension. This Frontiers Research Topic brings together contributions from a diverse array of cognitive neuroscience to offer a snapshot of current research on the neural substrate of figurative language, and present a number of avenues for future research. The result is an interdisciplinary perspective on the differences between literal and figurative language and how the underlying neurobiological processes can be investigated.

Indeed, most investigations into the neural substrate of metaphor ultimately concern the relationship between literal and metaphorical meanings. In their excellent review paper, Vulchanova and colleagues outline the arguments for and against the continuity thesis that literal and metaphorical language comprehension recruits essentially the same processing mechanisms. Using autism as a lens through which to consider the issue, they review data that indicate dissociations in the comprehension of literal and figurative language within individuals with ASD. Ultimately, they suggest figurative language deficits in ASD stem from the difficulty these individuals have integrating contextual information to build the situation model.

One source of support for the idea that literal and metaphorical comprehension processes recruit distinct neural substrates is the increasingly contentious claim that the right cerebral hemisphere (RH) plays a crucial role in the comprehension of metaphor, but not literal language. Ianni and colleagues note that much of the data supporting this claim comes from the study of brain-injured patients that have employed sub-optimal tasks for assessing metaphor comprehension. They present a novel test with fine-grained sensitivity to participants' ability to understand both literal and metaphorical language. They present data from three patients to demonstrate (i) comparable impairment on literal and metaphorical language, (ii) greater impairment for metaphorical than literal language, and (iii) selective impairment on metaphorical language.

Addressing the issue of hemispheric specialization in healthy adults, Lai and colleagues examine functional neuroimaging data as participants read literal and metaphorical sentences with varying degrees of familiarity. They found that decreasing familiarity (i.e., increasing novelty) of both literal and metaphorical language led to greater activation bilaterally, with more extensive recruitment of LH brain regions overall. However, the *relative* contribution of the RH was greater for novel metaphors, as a result of reduced LH activation for novel literal language.

Faust and colleagues utilize network theory in their discussion of hemispheric specialization for metaphor comprehension. In particular, they suggest that the LH exhibits semantic rigidity, manifested by networks in which each node is connected to a small number of other nodes. Rigid networks are well suited for the rapid retrieval of conventional meanings, but ill-suited for creating meanings needed for novel metaphors. The RH exhibits semantic chaos, manifested by highly inter-connected networks that enable fast connections between semantically distant concepts. Although inter-connectivity facilitates the comprehension of novel metaphors, its pathological extreme can be seen in schizophrenia and accompanying thought disorder.

Mashal and colleagues examined brain activity as schizophrenics and age-matched controls read literal phrases, conventional metaphors, and novel metaphors. They find novel metaphors elicited greater activity in the RH precuneus and superior parietal lobule (SPL) among schizophrenics than controls, and that greater activation in this brain region was correlated with better comprehension. In keeping with Faust and Kennet's suggestion that schizophrenia is associated with greater inter-connectivity in the semantic network, Mashal and colleagues found patients showed a greater degree of functional coupling between the precuneus/SPL and other language regions.

As is typical of studies of metaphor comprehension in schizophrenia, Mashal and colleagues found evidence for reduced comprehension in patients relative to controls. However, figurative language is diverse, and requires multiple mechanisms for its comprehension. Cognizant of this fact, Pesciarelli and colleagues investigated whether patients with schizophrenia can utilize both combinatorial mechanisms and the retrieval of stored meanings in their comprehension of idioms. They report evidence suggesting that the difficulty schizophrenic patients have understanding metaphors is less pronounced in the case of idioms for which they can rely on the retrieval of stored meanings.

Differences between the processing of metaphors and idioms are also supported by studies of healthy adults. Columbus and colleagues asked whether domain-general aspects of executive control influenced reading times for familiar and unfamiliar metaphorical sentences and idioms. They found that individuals with high executive control utilized context more efficiently than those with low executive control to commit to a metaphorical or literal reading of a target word. While executive control led to advantages for both familiar and unfamiliar metaphors, all participants read idioms efficiently, reinforcing the importance of retrieval mechanisms for idiom comprehension.

Metaphor comprehension is also impacted by individual differences in abstraction ability. Roncero and de Almedia examined figurative language processing in individuals with Alzheimer's disease (AD). In two studies they explored whether patients' abstraction ability was related to how well they interpreted metaphors, and whether saliency as measured by aptness ratings as well as familiarity ratings influenced patients' metaphor interpretation scores. Their findings suggest that patients with better abstraction ability produced better interpretations, and that aptness, not familiarity predicted patients' metaphor interpretation ability.

Other investigators have examined the importance of concreteness on understanding metaphors. Forgács and colleagues compared ERPs to nouns in phrases that were metaphorical (*fluffy speech*), concrete literal (*nasal speech*), and abstract literal (*ineffective speech*). Whereas, adjectives in literal phrases elicited typical ERP concreteness effects, nouns in metaphorical phrases did not. Paradoxically, when the novel metaphorical phrases were rated more concrete, the ERPs to the target nouns resembled those to nouns in abstract literal phrases. When the novel metaphorical phrases were rated more abstract, the ERPs to target nouns resembled those to nouns in concrete literal phrases. Results are argued to support a model in which the literal meaning from the concrete source domain in a metaphor is abstracted away from its physical sense before it is mapped to the abstract target domain.

Weiland and colleagues examined the priority of literal meaning during the processing of metaphor and metonymy. In this study, the literal meaning of the target expressions (e.g., *These lawyers are hyenas*) was induced via a briefly presented prime (*furry*) prior to the target (*hyenas*). At the target word they observed a reduction in the early ERP effect for both metaphors and metonymies and a delay in the late ERP effect only for metaphors. They suggested that the induced literal meaning facilitated the first stage of metaphor comprehension, which involves its literal sense.

Lakoff, in a theoretical contribution, outlined in brief his influential approach to metaphor, describing the existence of systematic metaphors in language, and arguing that they reflect regularities in the conceptual system, that are in turn driven by experiential correlations encoded neurally via spike timing dependent plasticity. Lakoff details empirical support for his theory that stems from linguistics, psychology, and cognitive neuroscience. He dispels some unflattering and oversimplified readings of his account, and sketches the beginnings of a neural theory of metaphor.

Lakoff's call for further research is taken up by a number of other contributors to the volume. These researchers present original research testing some of the predictions of embodied metaphor theory, and its more general counterpart in embodied cognition. For example, Schmidt-Snoek and colleagues compared the event related brain response to metaphors whose source domain evoked the auditory modality, as in “Her limousine was a privileged snort,” or the motor modality, as in “The editorial was a brass-knuckle punch,” and literal uses of the same words. They found that auditory words elicited a slightly different pattern of ERPs than did motor words, suggestive of non-overlapping neural generators. These findings fit with predictions from embodied metaphor theory that modality specific activations contribute to both literal and metaphoric meanings of these words.

Troyer and colleagues examine whether videos and still images of point light walkers impact reading times for sentences with action verbs. As one might expect from embodied models of cognition, the perception of visual motion does impact the processing of sentences with action verbs, but does so differently for literal uses, such as “The chemist was walking to his lab,” and metaphorical ones, such as “The company was walking to its death.”

Similarly, Bardolph and Coulson recorded ERPs as participants read words associated with different regions of vertical space as they moved marbles in an upward or a downward directed motion. Words such as “floor” and “ceiling” elicited very early congruency effects in the ERPs, consistent with the involvement of motor regions in registering the conflict between meaning and motion. Words such as “defeat” and “victory,” whose vertical associations were metaphorical, elicited congruency effects that emerged much later, in keeping with a role in pragmatic inference.

By focusing on novel research on the neural basis of metaphor, this Frontiers Research Topic provides insight into the neurobiology of conceptual structure. The contributions highlight how metaphor comprehension reveals hemispheric differences in the organization of semantic memory, the importance of executive function for high-level language comprehension, and the differing roles of sensorimotor activations for concrete and abstract concepts. Beyond the individual contributions, we hope that this special focus will inspire future research on the neural underpinnings of metaphor in language, and metaphor in cognition more generally.

## Author contributions

Both authors contributed equally to the writing and editing of this review article.

## Funding

SC received support from the Kavli Institute for Brain & Mind, San Diego.

### Conflict of interest statement

The authors declare that the research was conducted in the absence of any commercial or financial relationships that could be construed as a potential conflict of interest.

